# Biomechanical comparative study on external fixators of new configurations in the treatment of Tile C pelvic injury

**DOI:** 10.1038/s41598-024-60341-x

**Published:** 2024-04-25

**Authors:** Yong Zhao, Yupeng Ma, Hao Wu, Wei Lian, Wenliang Li, Wenkang Jiang

**Affiliations:** 1 Orthopaedics Department, Yantai Shan Hospital, 91#, Jiefang Road, Yantai, 264001 Shandong People’s Republic of China; 2 Yantai Key Laboratory for Repair and Reconstruction of Bone & Joint, Yantai Shan Hospital, 91#, Jiefang Road, Yantai, 264001 Shandong People’s Republic of China; 3 CT/MR Department, Yantai Shan Hospital, 91#, Jiefang Road, Yantai, 264001 Shandong People’s Republic of China

**Keywords:** Pelvis, Fracture, External fixator, Mechanics, Finite element analysis, Trauma, Fracture repair

## Abstract

To compare the biomechanical properties of several anterior pelvic ring external fixators with two new configurations in the treatment of Tile C pelvic fractures, in order to evaluate the effectiveness of the new configurations and provide a reference for their clinical application. A finite element model of a Tile C pelvic ring injury (unilateral longitudinal sacral fracture and ipsilateral pubic fracture) was constructed. The pelvis was fixed with iliac crest external fixator (IC), anterior inferior iliac spine external fixator (AIIS), combination of IC and AIIS, combination of anterior superior iliac spine external fixator (ASIS) and AIIS, and S1 sacroiliac screw in 5 types of models. The stability indices of the anterior and posterior pelvic rings under vertical longitudinal load, left–right compression load and anterior–posterior shear load were quantified and compared. In the simulated bipedal standing position, the results of the vertical displacement of the midpoint on the upper surface of the sacrum are consistent with the displacement of the posterior rotation angle, and the order from largest to smallest is IC, AIIS, ASIS + AIIS, IC + AIIS and S1 screw. The longitudinal displacement of IC is greater than that of the other models. The displacements of ASIS + AIIS and IC + AIIS are similar and the latter is smaller. In the simulated semi-recumbent position, the vertical displacement and posterior rotation angle displacement of the midpoint on the upper surface of the sacrum are also consistent, ranking from large to small: IC, AIIS, ASIS + AIIS, IC + AIIS and S1 screw. Under the simulated left–right compression load state, the lateral displacements of the highest point of the lateral sacral fracture end are consistent with the highest point of the lateral pubic fracture end, and the order from large to small is S1 screw, IC, AIIS, ASIS + AIIS and IC + AIIS, among which the displacements of S1 screw and IC are larger, and the displacements of ASIS + AIIS and IC + AIIS are similar and smaller than those of other models. The displacements of IC + AIIS are smaller than those of ASIS + AIIS. Under the simulated anterior–posterior shear load condition, the posterior displacements of the highest point of the lateral sacral fracture end and the highest point of the lateral pubic fracture end are also consistent, ranking from large to small: IC, AIIS, ASIS + AIIS, IC + AIIS and S1 screw. Among them, the displacements of IC and AIIS are larger. The displacements of ASIS + AIIS and IC + AIIS are similar and the latter are smaller. For the unstable pelvic injury represented by Tile C pelvic fracture, the biomechanical various stabilities of the combination of IC and AIIS are superior to those of the external fixators of conventional configurations. The biomechanical stabilities of the combination of ASIS and AIIS are also better than those of the external fixators of conventional configurations, and slightly worse than those of the combination of IC and AIIS. Compared with sacroiliac screw and conventional external fixators, the lateral stabilities of IC + AIIS and ASIS + AIIS are particularly prominent.

## Introduction

Vertical shear Tile C pelvic fractures result from major trauma, with axial loads displacing the hemipelvis causing injury to both anterior and posterior rings^1^. Compared to the stationary sacrum, displacement of the hemipelvis carries the risk of neurological and vascular injury, especially to the presacral plexus^2^. In a severely displaced injury, the pelvic volume expands and holds more blood. As a result, bleeding may be uncontrolled and life-threatening. Therefore, vertical and lateral displacement must be corrected to reduce the pelvic volume and facilitate bleeding control. In addition, early and adequate pelvic fracture reduction and fixation is essential to optimize the long-term prognosis of the patient, and conversely, is a risk factor for poor functional, chronic pelvic pain, deformity, gait disturbance, and significantly reduced quality of life^3^.

Because of early reduction of pelvic volume and stabilization of the pelvis, temporary external frame fixation is a relatively effective treatment, which helps the patient maintain a relatively upright position, optimizes cardiopulmonary physiology, improves mobility and flexibility, and improves care while awaiting final internal fixation, and has been successfully used in other types of pelvic fractures except Tile C^4^.

The two most common locations for external fixator Shanz pins are the iliac crest and the supra-acetabular bone, namely the external fixator of the iliac crest (IC) and the external fixator of the anterior inferior iliac spine (AIIS).

Several clinical studies have reported re-displacement after using external pelvic fixation, and in general, AIIS pelvic external fixation is superior to IC external fixation^5^, but there is still a high rate of re-displacement in type C pelvic fractures^6–8^. A problem with the conventional AIIS design is that there is only one Shanz pin in each hemipelvis, and the theoretical possibility of the pin rotating within the supra-acetabula bone is its biomechanical defect. Thus, under vertical loading, the unstable hemipelvis can be displaced vertically by rotation of the contralateral AIIS pin. In order to maximize the function of the external pelvic fixation and effectively fix the least stable Tile C pelvic fracture, it is necessary to improve the AIIS and IC to take advantage of the strengths and avoid the weaknesses of each of the two external fixators to stabilize the pelvic ring.

Similarly, the authors designed the anterior superior iliac spine (ASIS) external frame and the AIIS external frame to form a new configuration of ASIS + AIIS external frame, and carried out finite element biomechanical tests on the two new configurations of anterior ring external fixation frame mode, and compared them with various external fixation frame configurations and sacroiliac screws of sacral 1 (S1), in order to clarify the stability of the mechanical properties of their anterior and posterior rings. It is hoped that the two new configurations of anterior ring external fixation frame can provide more stable pelvic fixation.

A review of the literature reveals that there have been finite element biomechanical studies of minimally invasive fixation of the anterior pelvic ring in recent years, but either the studies have been limited to Tile B pelvic fractures^9^, which are relatively stable, or they have focussed on modifications of minimally invasive internal fixation of the anterior ring^10^.

## Methods

### Establishment of finite element models

#### Establishment of pelvic finite element model

A three-dimensional finite element model of the pelvis was established and improved according to the modeling method of our series of studies, and the relevant parameters were referred to the relevant literature of this series of studies^11–14^.

In order to define the solid geometry of the pelvic bones, an anatomic pelvic model from CT data of a healthy woman (36 years old, 170 cm, 63 kg) was constructed. A 64-slice spiral CT (Philips) was used to obtain images of pelvis with a scan thickness of 1 mm. The CT data was imported into medical software (mimics 19.0) to construct the 3D model of pelvis by means of the image processing algorithms such as threshold segmentation, region grow and so on. The pelvis model in STL file format was then imported into the reverse engineering software Geomagic Studio 2013 (Geomagic, USA) to remove the noise shells and generate the pelvis model with the geometric characterization of single manifold structure. The geometric model was used as the basis for defining the geometric extents of cortical and trabecular bone of the pelvis. Four-node linear solid tetrahedral elements with an average edge length of 2 mm were used in Ansys 19.0 software to create an unstructured mesh of the trabecular bone. Triangle shell elements with a thickness of 2 mm were used to represent the cortical bone, surrounding the trabecular bone^15^. Tied conditions were assumed between the internal surface of the cortical bone and the surface of the trabecular bone. Young’s modulus and Poisson’s ratio were taken to be 150 N/mm^2^ and 0.2 for trabecular bone, and 18,000 N/mm^2^ and 0.3 for cortical bone^15^. The sacroiliac cartilage and interpubic disc were represented as continuum structure occupying the inter-space and mesh into hexahedron element. Because of their important roles in pelvic biomechanics and stability, sacroiliac ligament, sacrospinous ligament and saerotuberous ligament, etc. were incorporated and modeled as tension-only discrete axial connectors. The attachment points were ascertained by being mimesissed anatomy as closely as possible. The material properties and elements used in the models are available in Tables 1 and 2^15–17^.

**Table 1:** Model parameters of pelvic ligaments.

Ligament	K (N/mm)	Number of springs
Anterior and capsule	700	27
Posterior (inner layer)	1400	15
Interosseous	2800	8
Sacrospinous	1400	9
Saerotuberous	1500	15
Superior pubic	500	24
Arcuate pubic	500	24

**Table 2:** Model parameters of various kinds of material.

	Young’s modulus (MPa)	Poisson’s ratio	Element type
Cortical bone	18,000	0.3	Shell element
Trabecular bone	150	0.2	Tetrahedral element
Sacroiliac cartilage	1000	0.3	Hexahedral element
Interpubic disc	5	0.45	Hexahedral element
Screw and pin	114,000	0.3	Hexahedral element

The FE model was validated using in vitro data and the results of simulation studies, which showed great consistency with classical studies^17^.

The central sagittal plane of the right inferior and superior pubic branches and the central sagittal plane of the right sacral foramen were defined as fracture surfaces in the normal pelvis model to simulate Tile C type unilateral pelvic fracture. Penalty contact with a friction coefficient of 0.3 was applied between the interaction surfaces of fractures.

#### Finite element modeling of minimally invasive external fixations

**Figure 1:**
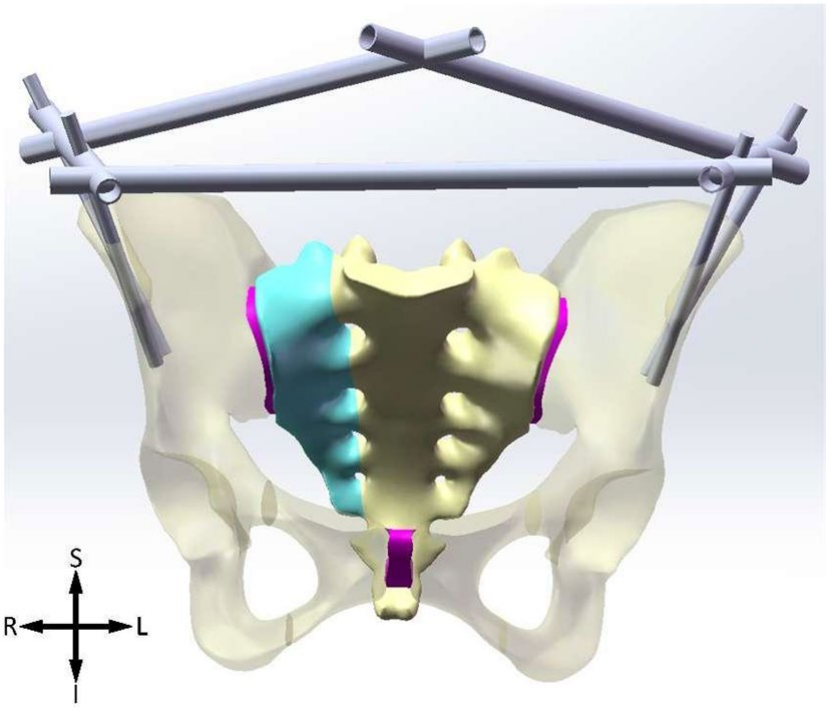
IC. *S* superior, *I* inferior, *L* left, *R* right.

**Figure 2:**
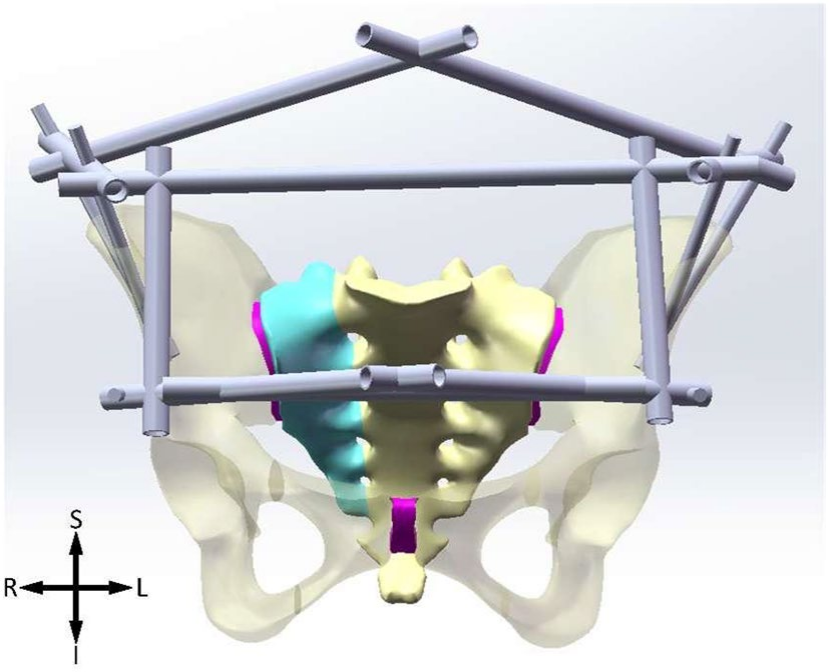
IC + AIIS. *S* superior, *I* inferior, *L* left, *R* right.

**Figure 3:**
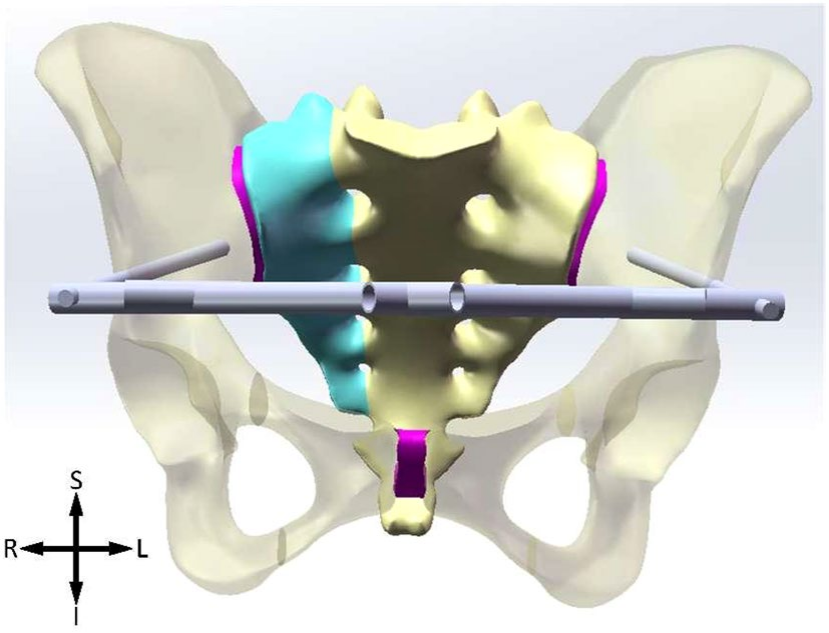
AIIS. *S* superior, *I* inferior, *L* left, *R* right.

**Figure 4:**
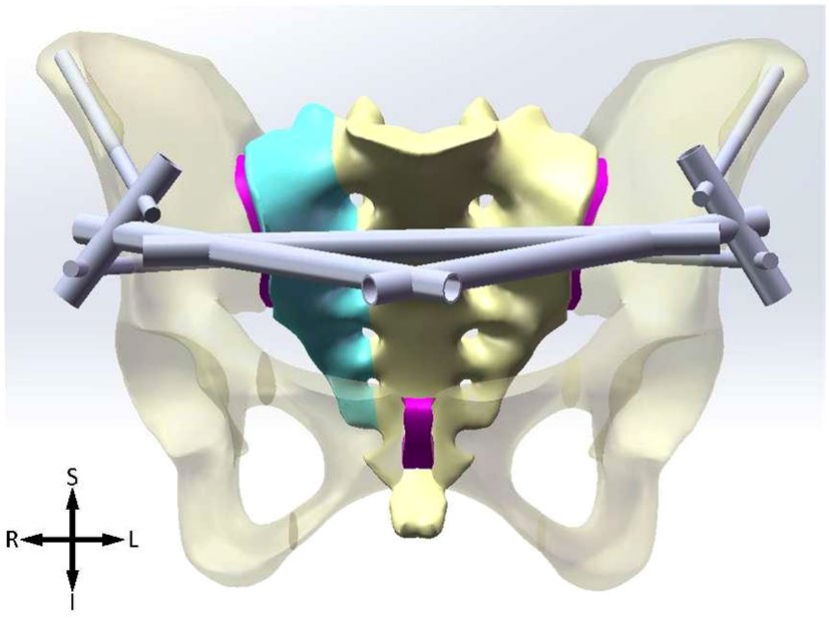
ASIS + AIIS. *S* superior, *I* inferior, *L* left, *R* right.

According to the dimensions of the Shanz pin and connecting rod, models was established by parametric design to simulate (1) external fixation of iliac crest (IC), (2) combination of external fixation of iliac crest and anterior inferior iliac spine (IC + AIIS), (3) external fixation of anterior inferior iliac spine(AIIS), and (4) combination of external fixation of anterior superior iliac spine and anterior inferior iliac spine (ASIS + AIIS) to fix the pelvis (Figs. 1, 2, 3, 4).

The Shanz pins are located between the inner and outer plates of the ilium, within the osseous safety zone, with a diameter of 6 mm and a length of 125 mm. The pins of IC are placed from the anterior iliac crest and point to the acetabulum. The ASIS pins are placed from the anterior superior iliac spine and travels along the broad iliac crest. The pins of AIIS are placed from the anterior inferior iliac spine and point to the posterior inferior iliac spine. The connecting rods, diameter 11 mm, their lengths are determined according to actual needs. The models of pins are subtracted from ilium to generate mounting holes, where a bonded contact boundary condition is applied to depict the connection relationship between pins and ilium.

#### Finite element modeling of sacroiliac screw fixation

**Figure 5:**
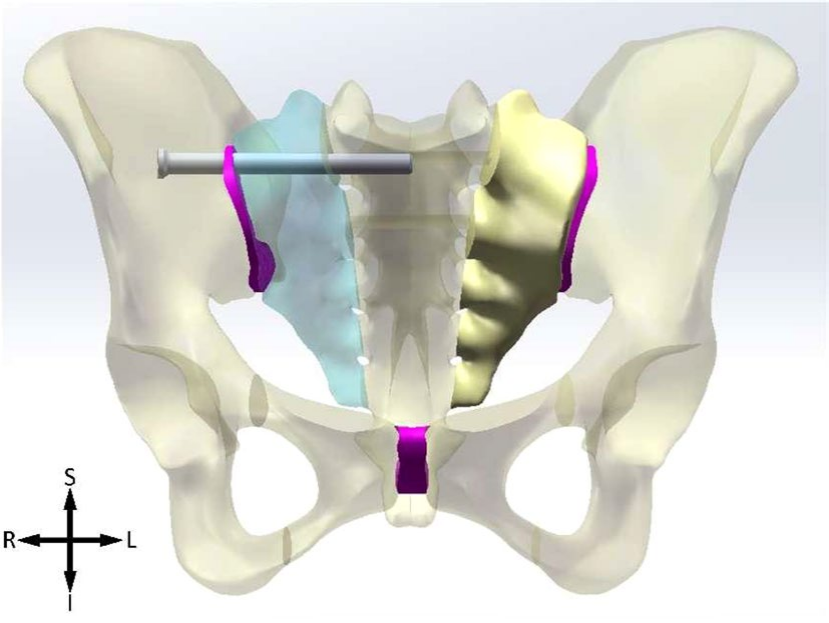
S1 screw. *S* superior, *I* inferior, *L* left, *R* right.

According to the sacroiliac screw dimensions, a parametric design was used to establish a model to simulate ordinary S1 sacroiliac screw (S1 screw) for fixation of the posterior pelvic ring (Fig. 5).

The sacroiliac screw is a 7.3-mm diameter cannulated screw that runs through the sacroiliac joint on the injured side via the S1 segment and is located within the bony safety zone of the posterior pelvic ring, traveling horizontally to the sacral midline, with a screw length of 85 mm. The models of screws are subtracted from ilium and sacrum to generate mounting holes, where a bonded contact boundary condition is applied to depict the connection relationship between screw and ilium or sacrum.

### Constraint and loading

Binding constraints were used between the sacrum and ilium and the sacroiliac joint, respectively, and between the bilateral pubic bones and pubic symphysis. Shanz pins and screws were bound to bone tissue to simulate complete osseointegration.

The following mechanical tests in three loading directions were simulated. First, the sacrum was simulated to be fixed and loaded vertically from the upper surface of the sacrum with 600 N and 300 N respectively. Second, the posterior aspect of the sacrum was simulated fixed, and the bilateral acetabulums were left–right loaded with 150 N from outside to inside simultaneously. Third, the posterior aspect of the sacrum was simulated fixed and the pelvis was loaded with 300 N on both sides of the anterior inferior iliac spine from anterior to posterior simultaneously.

### Mechanical analysis and evaluation indicators

Under vertical loading, the downward displacement and the angular displacement of the posterior rotation of the midpoint of the upper surface of the sacrum were measured and compared as indices of stability assessment. Under left–right loading, the inward displacements of the highest point of the lateral fracture ends of the sacrum and pubis were measured and compared as indices of stability assessment. The smaller the displacements, the better the stability. Under anterior–posterior loading, the posterior displacements of the highest point of the lateral fracture ends of the sacrum and pubis were measured and compared as indices of stability evaluation.

### Ethics approval and consent to participate

All methods were carried out in accordance with relevant guidelines and regulations. The ethics committee of Yantai Shan Hospital approved the study. Informed consents were obtained from individual participant included in the study.

## Results

The longitudinal pelvic loads were simulated by loading 600 N and 300 N vertically downward from the upper surface of the sacrum in the bipedal standing position and in the semi-recumbent position, respectively.

In the simulated bipedal standing position, the results of the vertical displacement of the midpoint on the upper surface of the sacrum are consistent with the displacement of the posterior rotation angle displacement, and the order from largest to smallest is IC, AIIS, ASIS + AIIS, IC + AIIS and S1 screw. The longitudinal displacement of IC is greater than that of other models. The values of longitudinal displacement and posterior rotation displacement of S1 screws are smaller than those of other models. The displacements of ASIS + AIIS and IC + AIIS are similar, and the latter are smaller (Table 3). In the simulated semi-recumbent position, the vertical displacement and posterior rotation angle displacement of the midpoint on the upper surface of the sacrum are also consistent, ranking from large to small: IC, AIIS, ASIS + AIIS, IC + AIIS and S1 screw. The displacements of IC, AIIS, ASIS + AIIS and IC + AIIS are relatively close. The displacements of S1 screws are smaller than those of other models (Table 4).

**Table 3:** Downward displacement and posterior rotational angular displacement of the upper surface of sacrum under vertical downward 600 N loading.

Different fixations	Downward translation (mm)	Posterior angle displacement (°)
IC	5.0794	2.7106
IC + AIIS	4.4571	2.6463
AIIS	4.8752	2.7050
ASIS + AIIS	4.6342	2.6644
S1 screw	2.1989	1.3050

**Table 4:** Downward displacement and posterior rotational angular displacement of the upper surface of sacrum under vertical downward 300 N loading.

Different fixations	Downward translation (mm)	Posterior angle displacement (°)
IC	2.6157	1.4586
IC + AIIS	2.3134	1.4022
AIIS	2.5335	1.4324
ASIS + AIIS	2.4041	1.4139
S1 screw	1.1621	0.7116

Under the simulated left–right compression load state, the lateral displacements of the highest point of the lateral sacral fracture end are consistent with the highest point of the lateral pubic fracture end, and the order from largest to smallest is S1 screw, IC, AIIS, ASIS + AIIS and IC + AIIS, among which the displacements of S1 screw and IC are larger, and the displacements of ASIS + AIIS and IC + AIIS are similar and smaller than those of other models. The displacements of IC + AIIS are smaller than those of ASIS + AIIS (Table 5).

**Table 5:** Lateral displacements of the highest points of the lateral sacral and pubic fracture ends under left–right 150 N loading.

Different fixations	Lateral displacement of the highest point of the lateral sacral fracture end (mm)	Lateral displacement of the highest point of the lateral pubic fracture end (mm)
IC	0.7859	6.8137
IC + AIIS	0.2292	1.6421
AIIS	0.3121	1.8245
ASIS + AIIS	0.2557	1.6724
S1 screw	0.8515	7.3892

Under the simulated anterior–posterior shear loading condition, the backward displacements of the highest point of the lateral sacral fracture end and the highest point of the lateral pubic fracture end are also consistent, ranking from large to small: IC, AIIS, ASIS + AIIS, IC + AIIS and S1 screw. Among them, the displacements of IC and AIIS are larger. The displacements of ASIS + AIIS and IC + AIIS are similar and the latter are smaller (Table 6).

**Table 6:** Backward displacements of the highest points of the lateral sacral and pubic fracture ends under anterior–posterior 300 N loading.

Different fixations	Backward displacement of the highest point of the lateral sacral fracture end (mm)	Backward displacement of the highest point of the lateral pubic fracture end (mm)
IC	9.8146	7.6633
IC + AIIS	6.3858	4.2803
AIIS	8.1627	4.7013
ASIS + AIIS	6.7869	4.6094
S1 screw	0.1046	0.9314

## Discussion

Vertical shear pelvic injury typically results from high-energy trauma which results in an absolutely unstable Tile C fracture. Minimally invasive fracture reduction and fixation within a short time after the injury is essential to control bleeding and optimize the patient’s long-term quality of life. In cases of multiple injuries or hemodynamic instability, whether laparotomy, closed thoracic drainage, angiographic embolization or open tamponade hemostasis is required, it needs to be performed in the supine position. Therefore, temporary or definitive rapid, minimally invasive external fixation in the supine position is an attractive option. Currently, external fixation is able to achieve relatively stable fixation for Tile B pelvic fractures, but the technique has limited performance in controlling vertical and rotational displacement of the hemipelvis for Tile C pelvic injuries^18^.

At present, the more widely used external pelvic fixation is the hybrid external fixation with better assembly and adjustment performance, and according to the location of the Shanz pin, the anterior ring external fixation is mainly divided into external fixation of iliac crest (IC) and external fixation of anterior inferior iliac spine (AIIS). Although the diameter and number of Shanz pins and the distance between the connecting rod and the bone can affect the fixation of the external fixation, the morphology of the bone, the degree of injury, the combined injuries and patients’ lifestyle can all contribute to the failure to achieve the desired stability of the pelvic ring. In order to increase the stabilizing effect of the external fixation, scholars in the early years conducted biomechanical studies on the configuration of the anterior ring external fixation and discarded the less stable rectangular and trapezoidal external configuration and suggested the triangular configuration with better fixation effect. However, there is still much room for progress in increasing the effectiveness of the external fixation.

The Shanz pin placement site of IC is superficial, simple to perform, and easy for junior doctors to carry out, but the fixation effect is average. Although AIIS has a deeper pin placement site and requires a higher surgical technique, the fixation is relatively firm and has become the first choice for anterior ring external fixation. However, as mentioned previously, the conventional AIIS with one pin in each of the bilateral supra-acetabular regions makes it difficult to control the rotational displacement in the coronal plane that may result from vertical loading. If two separate fixation points are provided in the hemi-pelvis to form a framework with multiple support points, rotational displacement of the Shanz pin can theoretically be prevented. Therefore, the present study was designed to add an anteroposterior pin (named ASIS external fixation in this paper) to AIIS, starting from the anterior superior iliac spine and running along the broad iliac crest on both the healthy and injured hemipelvic sides, to prevent rotational displacement of the stable hemipelvic pin in the coronal plane on the one hand, and to increase the control of the injured hemipelvis on the other hand, thus counteracting vertical displacement. In addition, this study combined IC with AIIS in order to find an effective means of stabilizing the pelvis by creating a three-dimensional configuration with horizontal and coronal pins.

Our previous study showed that the tendency of posterior ring displacement in Tile C pelvic fractures under vertical loading was mainly in the form of downward displacement in the longitudinal direction and posterior rotation angle displacement in the sagittal plane^11–13^. On this basis, this study investigates the effects of various external fixations on the stability of the posterior pelvic ring in bipedal standing and semi-recumbent positions, and clarifies the biomechanical characteristics of different configurations of external fixations by comparison. Since the posterior pelvic ring contributes approximately 60% of the overall pelvic stability, it is challenging to judge the biomechanical performance of multiple external fixation configurations via the anterior ring by comparing posterior ring stability indexes. Therefore, although the displacements of some models do not differ much, they still show the differences in biomechanical properties of various fixation methods.

This study shows that IC + AIIS and ASIS + AIIS in the bipedal standing position better demonstrate the advantages over other external fixation configurations against vertical loads than in the semi-recumbent position. The combination of ASIS and AIIS reduces the downward displacement and posterior rotation displacement in the AIIS model, which may be related to the anti-rotation effect of the ASIS Shanz pins. Similarly, the combination of AIIS and IC reduces the vertical downward displacement and posterior rotation displacement of the posterior ring in the IC model, and similarly reduces the downward displacement and posterior rotation displacement of the posterior ring in the AIIS model. Since the iliac crest is occupied by two Shanz pins of IC, IC + AIIS cannot be aided by the additional pin of ASIS. Thus the excellent biomechanical properties of the IC + AIIS configuration are related to the IC of two pins increasing the anti-rotation effect of the AIIS in the coronal plane, on the one hand, and to the stabilization mechanism of the IC + AIIS integrating the stability of the pins in the coronal and horizontal planes on the other hand. This helps to explain, to some extent, the superior mechanical properties of IC + AIIS over ASIS + AIIS and suggests that the role of the IC should be reassessed and clinically applied.

It is worth noting that this study shows that the IC + AIIS combination and ASIS + AIIS combination still perform well in the external fixation group under anteroposterior loads, and are the best two configurations among the external fixations in the stability comparison. The lateral stability performance of the external fixations was better than that of the sacroiliac screw under lateral compression load, with IC + AIIS and ASIS + AIIS performing more prominently. These suggest that the combination and modification of the external fixation configurations increases the pelvic left–right and anteroposterior stability, facilitating the patient’s postoperative side lying and turning, facilitating care and reducing complications. The results of this study showed that under the simulated semirecumbent loading, the posterior ring of the external frame fixation was less displaced and closer to the S1 sacroiliac screws, among which IC + AIIS and ASIS + AIIS were still preferred. This suggests that the new configuration of external fixation frame via the anterior pelvic ring can solve the problem of partial sitting up in the immediate postoperative period, thus facilitating the functional recovery of patients and reducing the incidence of pelvic fracture complications. These are the advantages of fixing the posterior pelvic ring to the pelvis as a whole via the external frame of the anterior pelvic ring. The authors have reason to believe that for Tile C1 pelvic fractures, the new configuration of the anterior ring external fixation frame can meet the emergency and first aid needs of controlling pelvic volume and reducing bleeding, as well as solving the postoperative semirecumbent position problem. Thus, in this type of injury, the overall stability of the pelvic ring can be quickly and minimally invasively partially restored by the simple and safe new configuration anterior ring external fixation, thus largely reducing the need for temporary and definitive fixation of complex and dangerous posterior pelvic ring injuries.

It should be noted that because of the complete disruption of the bilateral sacro-pelvic anatomic relationship in Tile type C2 and C3 pelvic fractures, anterior ring fixation alone cannot provide longitudinal stabilization between the sacrum and pelvis, so this study is only applicable to unilateral type C pelvic injuries (Tile type C1), i.e., to maximize stabilization of the affected posterior ring injured structures and even the whole pelvis by the more intact pelvic structures on the healthy side and fixation via the anterior ring. In addition, our previous biomechanical studies^11–14^ on sacral fractures and pubic fractures in the bipedal standing position found that the displacement of the intact pelvis was significantly less than that of the pelvic structures that were fixed after the injury. That is, any of the anterior (posterior) ring internal fixation complexes were less stable than the normal intact pelvis. Therefore, in order to minimize loss of reduction, we should try to avoid premature postoperative weight bearing on the affected part, even with the strongest modified external fixation.

Both classic external fixations, IC and AIIS, do not interfere with the abdominal surgery and their osseous fixation pathways do not interfere with each other. The stability performances of IC + AIIS and ASIS + AIIS are similar, and the placement of IC or ASIS pins on the basis of the AIIS does not significantly increase surgery injury and time due to the shallow entry points and relatively low difficulty of pins placement. We recommend the flexible use of one of two combinations to maximize the external fixation effect while safely and quickly installing the external fixation.

With the increasing depth and comprehensiveness of the research in all areas, it has to be pointed out that there are certain limitations in this study, which may be manifested in the following areas.

Patel et al.^19^ has proven that there is a contribution of the coating to implant stability. Travascio et al.^20^ documented that the bone can be seen as a porous media that consolidates during insertion of the pins. These should be taken into account in the modelling of further studies. While the focus of this study is on the mechanical properties of the external fixator, imaging-related factors require equal attention to maximise stability while maintaining a clinically acceptable level of MRI quality^21,22^. Additional limitation of the study is given by the fact that single lengths for the screws were used while studies performed by Morandi et al.^23^ and Young et al.^24^ have proven that for these insertion points multiple trajectories and pin lengths are safe to be used.

## Conclusions

For the unstable pelvic injury represented by Tile C pelvic fracture, the biomechanical various stabilities of the combination of IC and AIIS are superior to those of the external fixators of conventional configurations. The biomechanical stabilities of the combination of ASIS and AIIS are also better than those of the external fixators of conventional configurations, and slightly worse than those of the combination of IC and AIIS. Compared with sacroiliac screw and conventional external fixators, the lateral stabilities of IC + AIIS and ASIS + AIIS are particularly prominent.

## Data Availability

The datasets generated or analysed during the current study are not publicly available due the finite element model belongs to the subject but are available from the corresponding author on reasonable request.
